# Xylan oligosaccharides and cellobiohydrolase I (*Tr*Cel7A) interaction and effect on activity

**DOI:** 10.1186/1754-6834-4-45

**Published:** 2011-10-31

**Authors:** Martin J Baumann, Kim Borch, Peter Westh

**Affiliations:** 1 Research Unit for Biomaterials, Roskilde University, NSM, Universitetsvej 1, DK-4000, Roskilde, Denmark; 2 Novozymes A/S, Krogshøjvej 36, DK-2880, Bagsværd, Denmark

**Keywords:** cellobiohydrolase 1, *Tr*Cel7A, xylan, xylan oligosaccharide, binding, inhibition, biomass degradation, isothermal titration calorimetry

## Abstract

**Background:**

The well-studied cellulase mixture secreted by *Trichoderma reesei *(anamorph to *Hypocrea jecorina*) contains two cellobiohydolases (CBHs), cellobiohydrolase I (*Tr*Cel7A) and cellobiohydrolase II (*Tr*CeI6A), that are core enzymes for the solubilisation of cellulose. This has attracted significant research interest because of the role of the CBHs in the conversion of biomass to fermentable sugars. However, the CHBs are notoriously slow and susceptible to inhibition, which presents a challenge for the commercial utilisation of biomass. The xylans and xylan fragments that are also present in the biomass have been suggested repeatedly as one cause of the reduced activity of CHBs. Yet, the extent and mechanisms of this inhibition remain poorly elucidated. Therefore, we studied xylan oligosaccharides (XOSs) of variable lengths with respect to their binding and inhibition of both *Tr*Cel7A and an enzyme variant without the cellulose-binding domain (CBM).

**Results:**

We studied the binding of XOSs to *Tr*Cel7A by isothermal titration calorimetry. We found that XOSs bind to *Tr*Cel7A and that the affinity increases commensurate with XOS length. The CBM, on the other hand, did not affect the affinity significantly, which suggests that XOSs may bind to the active site. Activity assays of *Tr*Cel7A clearly demonstrated the negative effect of the presence of XOSs on the turnover number.

**Conclusions:**

On the basis of these binding data and a comparison of XOS inhibition of the activity of the two enzyme variants towards, respectively, soluble and insoluble substrates, we propose a competitive mechanism for XOS inhibition of *Tr*Cel7A with phosphoric swollen cellulose as a substrate.

## Background

Biomass is the most abundant raw material in the biosphere, and enzymatic biomass conversion of sugars and subsequent fermentation to transportation fuels has attracted enormous scientific and industrial interest [1]. In typical biomass raw materials such as corn stover or wheat straw, cellulose is not present in pure form but is in close contact with hemicelluloses such as xylan, mannan and xyloglucan and encrusted with lignin [2]. Many thermochemical pretreatments have been developed to disperse and dissolve the recalcitrant complex to make it more easily accessible for enzymatic hydrolysis [3].

Several phenomenological studies have documented a negative influence of xylans and xylan oligosaccharides (XOSs) on the performance of industrial cellulase mixtures [4-7]. However, which enzymes of the commercial cellulases are affected remains unresolved. Furthermore, target sites and mechanisms of these effects remain to be investigated. Such information will clearly be needed when considering remedies for xylan inhibition.

The term 'cellulase' most often refers to secreted cellulolytic enzymes of fungal origin. For instance, the cellulases secreted by *Trichoderma reesei *(anamorph to *Hypocrea jecorina*) contain several endoglucanases and two cellobiohydrolases (CBHs) which process *exo*-acting, cellobiose-releasing enzymes. The CBHs exhibit quite low reaction rates and non-Michaelis-Menten kinetics, which include an initial burst followed by a biphasic slowdown [8,9]. Classical inhibition studies which result in an inhibition constant *K*_i _are not applicable to cellobiohydrolases, because the concentration of the insoluble substrate cellulose is very difficult to measure and, as mentioned above, steady-state reaction kinetics are absent. In industrial batch hydrolysis of biomass CBHs can suffer from product inhibition, which is partially reduced through the cellobiase activity of added β-glucosidases [9,10]. Traditionally, CBHs have been claimed to require free chain ends as starting points and free cellulose chain ends are in turn generated by endoglucanases, which hydrolyse the cellulose chain randomly in *endo*-fashion. However, this strict classification has been challenged by the demonstration of *endo*-attack by CBHs and processive action of endoglucanases [11].

Cellobiohydrolase I (*Tr*Cel7A) is the most abundant enzyme secreted by *Trichoderma reesei*. It is a modular 52 kDa enzyme with a catalytic domain (GH7) [12] and a family 1 cellulose binding domain (http://www.cazy.org/), which are interconnected by a highly glycosylated linker [13,14]. The catalytic domain has a tunnel-shaped active site with ten subsites [14].

In this study, we investigated the apparent inhibition of *Tr*Cel7A by xylan products in a model system with XOSs, which are enzymatically derived from birch wood xylan. *Tr*Cel7A is used in two variants, the full-length enzyme (*Tr*Cel7A) and the catalytic domain only (*Tr*Cel7A core). The influence of the degree of polymerisation (DP) of XOS ligand binding was tested by performing isothermal titration calorimetry (ITC). Activity measurements of TrCel7A were conducted with *para*-nitrophenyl-β-D-lacoside (*p*NP-lac) or phosphoric acid swollen cellulose (PASC) in the presence of XOS pools of different DPs.

## Results

**Figure 1:**
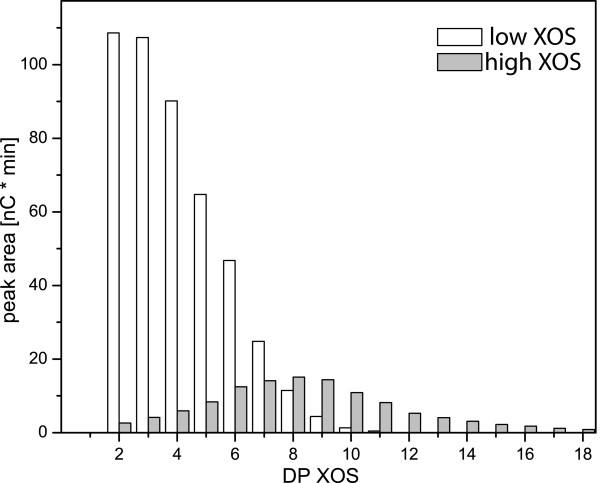
**HPAEC-PAD analysis of 1 g/L low xylan oligosaccharide and 1 g/L high xylan oligosaccharide**. High-performance anion-exchange chromatography with pulsed amperometric detection (HPAEC-PAD) analysis of low xylan oligosaccharide (XOS, open columns) and high XOS (grey columns). The peak from xylobiose to xylopentose was identified by comparison with commercially available XOSs, and longer oligosaccharides were named accordingly. The peak areas of 10 μL of 5 g/L solutions are shown. The response factors for xylobiose to xylopentose were between 0.09 and 0.1 and were almost constant. The average degree of polymerisation (DP) (by peak area) of low XOS was 4.0, 80% between (xyl)_2 _and (xyl)_6 _and 90% between (xyl)_2 _and (xyl)_7_; the average DP of high XOS was 8.1, 80% between (xyl)_5 _and (xyl)_13 _and 90% between (xyl)_3 _and (xyl)_13_.

The interaction of XOSs with *Tr*Cel7A was investigated using two approaches: ITC binding experiments and activity measurements. The activity measurements were carried out on PASC and/or *p*NP-lac in the presence of enzymatically prepared mixed XOSs. These measurements require large amounts of XOS (gram-scale) which were not available from the purification of XOSs by size exclusion chromatography. To investigate the influence of the average molecular mass, XOS was split into two fractions by ethanol precipitation. Low-molecular-mass XOS (low XOS) and high-molecular-mass XOS (high XOS) have an average of 4.0 or 8.1 DP xylose units, respectively (Figure 1).

We chose to assess *Tr*Cel7A activity in the presence of XOS using two different assay methods. First, a small chromogenic lactose, *p*NP-lac, was used in a straightforward stopped assay to assess the activity of *Tr*Cel7A [15]. *p*NP-lac activity has been used by Jalak and Välijamäe [16] to measure the portion of *Tr*Cel7A that has a free, unoccupied active site. The specific activity of *Tr*Cel7A for *p*NP-lac in our study was approximately a factor of 10 lower than that for PASC (Table 1).

**Table 1:** Specific activities of *Tr*Cel7A and *Tr*Cel7A core at 50°C per hour

Enzyme	Substrate	*k*_cat_
*Tr*Cel7A	PASC (1 g/L)	0.44/second
*Tr*Cel7A core	PASC (1 g/L)	0.24/second
*Tr*Cel7A	*p*NP-lac (0.5 mM)	0.07/second
*Tr*Cel7A core	*p*NP-lac (0.5 mM)	0.06/second

*k*_cat _= catalytic constant; PASC = phosphoric acid swollen cellulose; *p*NP-lac = *para*-nitrophenyl-β-D-lacoside; *Tr*Cel7A = cellobiohydrolase I.

We used a second approach to measure *Tr*Cel7A activity in which we quantified the cellobiose release from PASC by high-performance anion-exchange chromatography with pulsed amperometric detection (HPAEC-PAD). Typical Cel7A assays include cellobiase (for example, β-glucosidase) to avoid product inhibition, but many β-glucosidases show activity on nitrophenyl xyloside [17-19] and may also have activity on XOS, which might obfuscate the activity data of the current inhibition studies. To dissect the mechanism of *Tr*Cel7A-inhibition by XOS further, we used the modularity of the enzyme to test the influence of the CBM on activity in the presence of XOS. We used two variants of *Tr*Cel7A, the full-length enzyme and the *Tr*Cel7A core (*Tr*Cel7A truncated on DNA level by the linker and the CBM), which allowed us to study the influence of the CBM on XOS inhibition.

**Figure 2:**
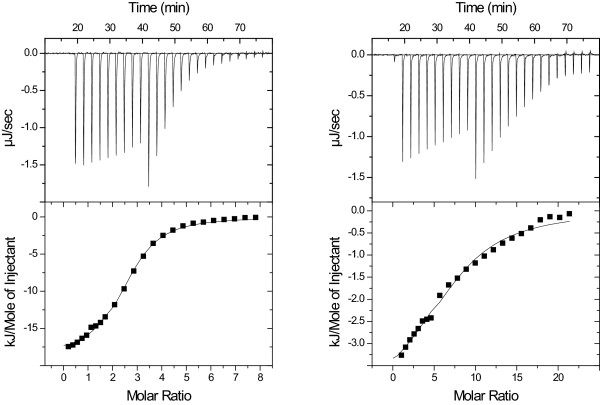
**Isothermal titration calorimetry experiments**. Upper panels: Isothermal titration calorimetry raw data and binding curve of cellobiohydrolase I (*Tr*Cel7A) with nine 1-μl injections of xylan oligosaccharide (XOS), followed by fifteen 2-μl injections of XOS. Lower panels: Integrated peak areas (squares) and the best fit of the model. All binding experiments were conducted at 30°C. Left: A 1:1 mixture of xylooctaose (xyl)_8 _and xylononaose (xyl)_9 _was injected into *Tr*Cel7A. Right: The high XOS mixture was injected into *Tr*Cel7A.

ITC isotherms at 30°C for both purified and mixed XOSs were readily analysed using standard binding models provided by the MicroCal/GE Healthcare, (Northampton, MA, USA) instrument manufacturer (Figure 2). At 50°C, the signals were small and the quality of the data did not allow regression analysis. This most likely reflects the binding enthalpies becoming smaller at the higher temperature, hence ITC was not a suitable experimental method. The binding constants at 30°C increased from about 10^3 ^for the trisaccharide to over 10^5 ^for a XOS mixture with a 1:1:1 ratio (8 DP, 9 DP and 10 DP) (Table 2). Table 2 also shows that both enzyme variants had similar affinity for XOS. To study the interaction of *Tr*Cel7A and XOS, we measured the binding of pure linear XOSs to *Tr*Cel7A by ITC. The binding constants increased strongly with chain lengths of the XOS (Table 1). A typical example of the binding isotherms obtained is shown in Figure 2.

**Table 2:** Binding constants based on a molar concentration scale of *Tr*Cel7A and *Tr*Cel7A core to linear xylan oligosaccharides

Xylan oligosaccharide	*Tr*Cel7A core (*K*)	*Tr*Cel7A (*K*)
Xylotriose (xyl)_3_	> 1.0 × 10^3^	-
Xylotetraose (xyl)_4_	2.97 × 10^3^	-
Xylohexaose (xyl)_5_	3.44 × 10^3^	3.44 × 10^3^
Xyloheptaose (xyl)_7_	7.43 × 10^3^	5.76 × 10^3^
Xylooctaose/xylononaose, (xyl)_8_/(xyl)_9 _(1:1)	1.19 × 10^4^	9.20 × 10^4^
Xylooctaose/xylononaose/xylodecaose, (xyl)_8_/(xyl)_9_/(xyl)_10 _(1:1:1)	2.91 × 10^5^	-
Low XOS (5 g/L and 8.4 mM, average 4 DP)^a^	7.18 × 10^2^	9.13 × 10^2^
High XOS (5 g/L and 4.4 mM, average 8 DP)^a^	4.57 × 10^3^	3.47 × 10^3^

DP = degree of polymerisation; *Tr*Cel7A = cellobiohydrolase I; XOS = xylan oligosaccharide; - = no data. ^a^Estimated on the basis of high-performance anion-exchange chromatography with pulsed amperometric detection traces and binding experiments (*n *= 3).

### TrCel7A hydrolytic activity on pNP-lac

**Figure 3:**
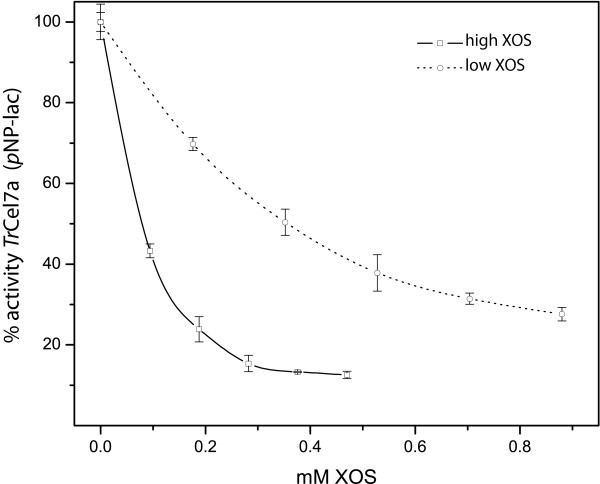
**Inhibitory effects of xylan oligosaccharide on the *para*-nitrophenyl-lac activity of *Tr*Cel7A**. The inhibitory effects of high xylan oligosaccharide (XOS) (open squares) and low XOS (open circles) on the *para*-nitrophenyl-lacoside (*p*NP-lac) hydrolytic activity of cellobiohydrolase I (*Tr*Cel7A) at 50°C are shown. The molarities based on the estimates measured by high-performance anion-exchange chromatography with pulsed amperometric detection analysis (see Figure 1), 0.5 g/L high XOS and 0.5 g/L low XOS, were estimated to be 0.47 mM and 0.88 mM, respectively. The error bars represent the standard deviations of three experiments.

*p*NP-lac was used as a small model substrate for Cel7A [14-16,20]. The effect of low XOS and high XOS was tested with *p*NP-lac as the substrate (Table 3). The addition of 1 g/L low XOS reduced the remaining activity to 21% or 15% for *Tr*Cel7A core or *Tr*Cel7A, respectively. The addition or 1 g/L high XOS removed 90% of the control *p*NP-releasing activity. In these experiments, the control activity *Tr*Cel7A and *Tr*Cel7A core did not differ substantially, indicating that the presence of the CBM had little or no effect on the activity on the soluble, small-molecule *p*NP-lac. Dose-response curves were generated for *Tr*Cel7A at 50°C (Figure 3). The curve for high XOS declined more sharply at lower concentrations than the inactivation curve of low XOS. This demonstrates the relatively greater importance of molecular length (high XOS vs low XOS) over the molecular concentrations of XOS.

**Table 3:** *Para*-nitrophenyl release of *Tr*Cel7A and *Tr*Cel7A core from *p*NP-lac as a single substrate at 50°C and 30°C^a^

Enzyme (temperature substrate)	Activity in 1 hour	Activity in 1 hour (1 g/L low XOS)	Activity in 1 hour (1 g/L high XOS)
*Tr*Cel7A core, 50°C	127 ± 5 μM *p*NP^b^ 436 ± 11 μM cb^b^	21% ± 4 *p*NP 61% ± 6 cb	10% ± 2 *p*NP 44% ± 2 cb
*Tr*Cel7A, 50°C	99 ± 3 μM *p*NP 695 ± 32 μM cb	15% ± 2 *p*NP 96% ± 4 cb	9% ± 1 *p*NP 81% ± 5 cb
*Tr*Cel7A core, 30°C	50 ± 2 μM *p*NP 177 ± 6 μM cb	21% ± 2 *p*NP 61% ± 7 cb	10% ± 4 *p*NP 55% ± 4 cb
*Tr*Cel7A, 30°C	38 ± 1 μM *p*NP 204 ± 10 μM cb	15% ± 3 *p*NP 96% ± 2 cb	9% ± 3 *p*NP 81% ± 2 cb

cb: cellobiose-solubilised; *p*NP = *para*-nitrophenyl release; *p*NP-lac = *para*-nitrophenyl-β-D-lacoside; *Tr*Cel7A = cellobiohydrolase I; XOS = xylan oligosaccharide. ^a^The cellobiose release from phosphoric acid swollen cellulose (PASC) in the presence of *p*NP-lac by *Tr*Cel7A and *Tr*Cel7A core at 50°C and 30°C was measured in separate experiments. The deviations are the standard deviations of five independent experiments. ^b^The cellobiose release from PASC was independent of *p*NP-lac (Table 4).

### Hydrolytic activity on PASC in the presence of pNP-lac

*Tr*Cel7A activities were evaluated with PASC in the presence of *p*NP-lac substrate (Table 3). Control experiments revealed that the presence on 1 mM *p*NP-lac changed the release of cellobiose by 10% (Table 4), which for most of the samples was within the standard deviation. The specific activity of *Tr*Cel7A on PASC was twice as high as that of *Tr*Cel7A core. (Table 1) The susceptibility to XOS inhibition of *Tr*Cel7A and *Tr*Cel7A core on PASC differed widely. Low XOS practically did not change the activity of *Tr*Cel7A, and *Tr*Cel7A core produced only 60% of the cellobiose in the presence of low XOS. High XOS reduced the activity of both tested *Tr*Cel7A variants. Full-length *Tr*Cel7A activity produced 80% of the cellobiose, and *Tr*Cel7A core activity was reduced to values close to 50% of the cellobiose produced without added XOS.

**Table 4:** Cellobiose release from phosphoric acid swollen cellulose by *Tr*Cel7A and *Tr*Cel7A core at 50°C and 30°C

Enzyme (temperature substrate)	Positive control	1 g/L low XOS	1 g/L high XOS
*Tr*Cel7A core, 50°C	457 ± 10 μM cb	260 ± 19 μM cb	196 ± 3 μM cb
PASC	100%	57%	43%
*Tr*Cel7A, 50°C	695 ± 65 μM cb	611 ± 60 μM cb	587 ± 57 μM cb
PASC	100%	88%	85%
*Tr*Cel7A core, 30°C	183 ± 7 μM cb	111 ± 8 μM cb	87 ± 5 μM cb
PASC	100%	61%	48%
*Tr*Cel7A, 30°C	194 ± 8 μM cb	194 ± 4 μM cb	196 ± 1 μM cb
PASC	100%	96%	100%

cb: cellobiose-solubilised; PASC = phosphoric acid swollen cellulose; *Tr*Cel7A = cellobiohydrolase I; XOS = xylan oligosaccharide. The deviations are the standard deviations of five independent experiments.

### TrCel7A hydrolytic activity on pNP-lac in the presence of PASC

The activity of unbound *Tr*Cel7A and *Tr*Cel7A core in the presence of PASC was followed by *p*NP-lac hydrolytic activity (Table 5). The *p*NP-lac hydrolytic activity of *Tr*Cel7A and *Tr*Cel7A core was, respectively, 7% and 20% of the total activity against *p*NP-lac observed in the absence of PASC (Table 5). This indicates that 93% or 80%, respectively, of the *Tr*Cel7A or *Tr*Cel7A core active sites are not available for *p*NP-lac hydrolysis. The addition of low XOS and high XOS further reduced *p*NP-lac hydrolytic activity. Again, as we observed with regard to cellulolytic activity on PASC, high XOS produced a greater reduction of *p*NP-lac activity than low XOS did. Because the *p*NP-lac activity of *Tr*Cel7A in the presence of PASC was already reduced to 7% of the initial *p*NP-lac activity, further reductions in *p*NP-lac activity induced by the addition of XOS could not be quantified. The fractional decrease in *p*NP-lac activity brought about by PASC was larger at 30°C than at 50°C, which indicates that more enzyme may be bound at the lower temperature.

**Table 5:** *Para*-nitrophenyl release by *Tr*Cel7A or *Tr*Cel7A core from *p*NP-lac at 50°C and 30°C in the presence of phosphoric acid swollen cellulose

Enzyme, temperature substrate	Control	1 g/L low XOS	1 g/L high XOS
*Tr*Cel7A core, 50°C *p*NP-lac^a ^(PASC)	25 ± 2 μM *p*NP 20% of total activity	15 ± 4 μM *p*NP 12% of total activity	9 ± 1 μM *p*NP 8% of total activity
*Tr*Cel7A, 50°C *p*NP-lac (PASC)	6 ± 1 μM *p*NP 7% of total activity	5 ± 1 μM *p*NP 5% of total activity	< 4 μM *p*NP^b^
*Tr*Cel7A core, 30°C *p*NP-lac (PASC)	6 ± 1 μM *p*NP 12% of total activity	< 4 μM *p*NP^b^	< 4 μM *p*NP^b^
*Tr*Cel7A, 30°C *p*NP-lac (PASC)	< 4 μM *p*NP^b^	< 4 μM^b^	< 4 μM *p*NP^b^

PASC = phosphoric acid swollen cellulose; *p*NP = *para*-nitrophenyl; *p*NP-lac = *para*-nitrophenyl-β-D-lacoside; *Tr*Cel7A = cellobiohydrolase I; XOS = xylan oligosaccharide. The percentages were calculated on the basis of the total *p*NP activity shown in Table 3. The deviations are the standard deviations of five independent experiments. ^a^The *K*_i _for cellobiose in the *p*NP-lac assay is 20 μM, which is exceeded here. The activities are underestimated. ^b^The detected absorbance changes were below 0.02 absThe activities were too low for reliable quantification.

## Discussion

We tested the effect of XOSs on the activity of two *Tr*Cel7A variants in samples with PASC, *p*NP-lac or both. The binding affinity of XOS with known DP was also quantified for both enzyme variants. The combined interpretation of the binding and activity data suggests that XOS binds to the active site of TrCel7A and hence exerts competitive inhibition. Moreover, our results suggest that the affinity for the active site follows the sequence PASC > XOS >*p*NP-lac and consequently that XOS is a much stronger inhibitor of *p*NP-lac hydrolysis than of PASC hydrolysis. Next we discuss arguments for and aspects of this proposed mechanism of XOS inhibition.

With *p*NP-lac alone (Table 3), the specific activities of *Tr*Cel7A and *Tr*Cel7A core were essentially the same and the addition of low XOS or high XOS resulted in severe and similar activity loss for both enzymes. Hence the CBM does not play a role for the small, soluble substrate analog *p*NP-lac. These observations and the strong affinity of XOS observed for both enzyme variants suggest that XOS bind more strongly than *p*NP-lac and hence inhibits their activity. The inhibition by XOSs was much less pronounced when PASC was used as the substrate. In fact, the intact enzyme was not detectably inhibited by low XOS (Table 4). This difference most likely reflects the higher binding affinity of PASC strands to the catalytic domain of *Tr*Cel7 compared to the binding of *p*NP-lac to the *Tr*Cel7A catalytic domain. An analogous relationship was measured with cellobiose as a competitive inhibitor. The reported *K*_i _value for cellobiose with *p*NP-lac is 20 μM [20], and for bacterial cellulose it is approximately 50 times higher at around 1 mM [21]. This hypothesis is also in line with the observation that high XOS, despite the lower molar concentration (1 g/L equals about 4 mM), was caused greater inhibition than low XOS (1 g/L equals about 8 mM) (Table 3 and Figure 3). This can be simply explained by the higher binding affinity (Table 2) of high XOS, which overcompensates the lower molarity of high XOS.

Using PASC as a substrate, we observed that the presence of the CBM in *Tr*Cel7A plays a more prominent role in the sensitivity to XOS inhibition. The *p*NP-lac measurements in the presence of PASC showed that at most 7% of the added *Tr*Cel7A had an accessible active site. Thus we concluded that the missing 93% *p*NP-lac activity is removed by binding of *Tr*Cel7A to PASC, followed by occupation of the active site by a cellulose strand. The *Tr*Cel7A variant without CBM showed an increased *p*NP-lac activity (20% of the added activity is free in the buffer, when compared with the full length enzyme. This indicates that the presence of the CBM decreases the probability of a free active site in the presence of PASC. Our results derived from the two measurements combined gives a strong indication of the mechanism of activity inhibition of *Tr*Cel7A by XOSs. In a hydrolytic reaction, more than 80% of *Tr*Cel7A is bound to the substrate (Table 5). The presence of the CBM changed the equilibrium between free and bound *Tr*Cel7A from 20% free to less than 10% free in our experiments. This shift was even more pronounced at 30°C than at 50°C. The presence of the family 1 CBM in *Tr*Cel7A plays a pivotal role in its location and thereby its sensitivity to inhibition by XOSs. As the location equilibrium is shifted towards the insoluble substrate, the sensitivity to XOS inactivation of *Tr*Cel7A decreases.

**Figure 4:**
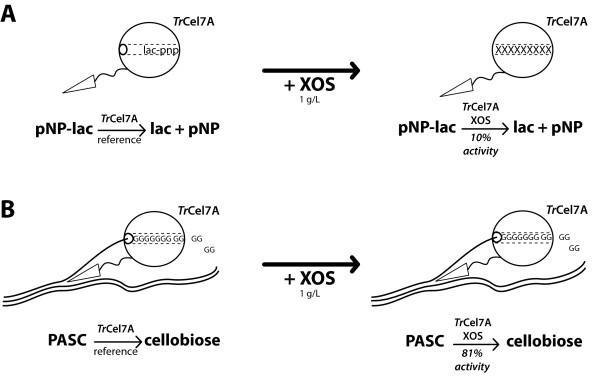
**Cartoon representing the inhibition mechanism of xylan oligosaccharide with different substrates**. **(A) **Xylan oligosaccharide (XOS) inhibition mechanism with *para*-nitrophenyl-lacoside (*p*NP-lac) used as the substrate. **(B) **XOS inhibition mechanism with PASC used as the substrate. In both cases, the activity of cellobiohydrolase I (*Tr*Cel7A) was decreased by the presence of XOS. The active site was most likely occupied by unproductively bound XOS, preventing the substrate from entering the active site tunnel. The degree of inhibition is dependent on the ratio of the binding affinities between the substrate and the inhibitor.

The results of our ITC experiments (Table 2) show that increasing the chain length of XOS is the main factor influencing binding affinity (Figure 4). The CBM seems not to interact with XOS, as the binding constant does not increase when the CBM is present. Interestingly, the binding of XOS observed by ITC also influenced the activity of *Tr*Cel7A on PASC, but to a certain extent PASC and XOS compete for binding. The activity data also confirmed the ITC binding data regarding the effect of the chain length, because high XOS reduced the activity of *Tr*Cel7A more than low XOS did. Additionally, temperature influenced the competition between XOS and PASC. At 30°C, the productive binding of *Tr*Cel7A to PASC was greater. At 50°C, the equilibrium shifted slightly towards the unproductive binding of XOS. On the basis of the measured binding enthalpies and the van 't Hoff equation, we predict that the binding constant for the XOS will decrease by about 40% when the temperature is increased from 30°C to 50°C. However, changes in the affinity to cellulose are unknown, and at this point we can only speculate about the balance between the individual binding constants at higher temperatures.

XOSs are linear oligosaccharides with the same type of β(1→4) bond as cellulose. The structural similarity between XOSs and cellulose may make it possible to fit XOS sterically into the active site of *Tr*Cel7A. The results of our ITC-binding experiments reveal that increasing the chain length of XOS promotes binding to *Tr*Cel7A. The highest binding constants were found for XOS, which approximately matched the length of the *Tr*Cel7A active site (Table 1). In our opinion, it is plausible that XOS could be a competitive inhibitor of *Tr*Cel7A by occupying the active site in place of the substrate. This interpretation is in accord with both the absence of a CBM effect in the binding studies and the presence of such an effect for PASC hydrolysis, but further (structural) studies are needed to unequivocally confirm this hypothesis.

In principle, all active cellobiose-releasing complexes are formed with *Tr*Cel7A recruited from the small free pool of enzyme in solution; therefore, XOS inhibition can play a major role in reducing *Tr*Cel7A activity. This study clearly shows that a higher incubation temperature of 50°C increases the XOS inactivation of *Tr*Cel7A.

## Conclusions

In biomass hydrolysis experiments, the addition of xylanases and/or xylosidases increases the overall yield of glucose and xylose [4,7,22,23]. The observed synergy between xylanases and cellulases can at least partly be explained by the offset of cellulase inhibition as XOS is hydrolysed to low DP, which does not bind strongly to the active site of cellulose. It remains to be discovered whether this is only the effect of the addition of xylanases or whether there are other substrate-related effects, too.

## Methods

### Xylan oligosaccharides

Mixed and pure XOSs were produced as described by Baumann *et al*. [24]. Monodisperse xylotriose to xyloheptaose was obtained in 100-mg amounts, and mixed pools were obtained up to xylodecaose as described in Table 3. High XOS and low XOS were separated by ethanol precipitation (4:1) of high XOS from a solution of mixed XOS (0.5 L at 14 g/L concentration) in 50 mM sodium acetate, pH 5.0. The fine precipitate (high XOS) was separated on a glass fibre filter and dissolved in water. After freeze-drying this solution, 2.1 g of low XOS and 4.6 g of high XOS were obtained. Both fractions were analysed by HPAEC-PAD (Figure 1). We found that low XOS contained saccharides from xylobiose to xylose_11 _with 80% of the relative peak area between xylobiose and xylopentose, whereas high XOS contained saccharides from xylobiose to xyl_18 _with 80% of the relative peak area between xylotetraose and (xyl)_12_. The average molecular mass values of low XOS and high XOS were calculated as 4.0 and 8.1 xylose units, respectively, after the relative peak areas had been determined.

### Enzymes

*Tr*Cel7A core was produced by site-directed mutagenesis of a *Tr*Cel7A sequence [UniProtKB:P62694]. The protein sequence of the catalytic domain used was the same sequence as that of *Tr*Cel7A but truncated by the carboxy-terminal CBM and the linker sequence. The mature *Tr*Cel7A core contained the first 436 amino acids after the signal peptides (17 amino acids). *Tr*Cel7A [UniProtKB:P62694] and *Tr*Cel7A core were both produced heterologously in *Aspergillus oryzae *by Novozymes A/S (Bagsværd, Denmark) according to patent WO/2000/039322 and were purified as described by Praestgaard *et al*. [9].

### ITC binding

The binding affinity of *Tr*Cel7A and *Tr*Cel7A core was measured at 30°C in an iTC_200 _power-compensated isothermal titration calorimeter (MicroCal/GE Healthcare, Northampton, MA, USA) with a cell volume of 200 μl and by using an injector equipped with a 40-μl syringe. All binding experiments were carried out at 30°C with 1,000 rpm stirring. In a typical binding experiment, the cell was filled with 40 μM cellobiohydrolase, and pure XOS (1.25 mM) or pools of mixed oligosaccharides (low XOS 5 g/L about 8.4 mM or high XOS 5 g/L about 4.4 mM) were injected according to the following protocol: initial equilibration for 16 minutes, then nine injections of 1 μl after 150 seconds, followed by fifteen injections of 2 μl. Data were analysed using Origin 7.0 software (OriginLab, Northampton, MA, USA) with the add-on provided by MicroCal/GE Healthcare.

### Enzyme activity

Cellobiohydrolase activity was measured as cellobiose release from 1 g/L PASC at 50°C or 30°C with 300 rpm shaking in an Eppendorf heating block. *Tr*Cel7A and *Tr*Cel7A core amounts were adjusted to release 100 μM or 127 μM *p*NP from *p*NP-lac when incubated at 50°C for one hour. After one hour of incubation, PASC was removed by short centrifugation for 20 seconds in a tabletop centrifuge. An aliquot of the supernatant (0.5 ml) was removed and incubated at 99°C for 20 minutes at 300 rpm in an Eppendorf heating block to inactivate the cellobiohydrolase. We analysed 10 μl of the supernatant by HPAEC-PAD. XOSs (200 μl) were added to a final concentration of 1 g/L, which is approximately equivalent to 1.7 mM low XOS or 0.9 mM high XOS. The amount of free cellobiohydrolase was quantified as *p*NP release from *p*NP-lac as described by Jalak and Väljamäe [16].

### Product inhibition of TrCel7A

A simple quantification of *Tr*Cel7A activity is hindered by several parameters. *Tr*Cel7A is product-inhibited by cellobiose. In the hydrolysis of bacterial cellulose, the product inhibition by cellobiose was measured by Gruno *et al*. [21] as *K*_i _approximately 1.5 mM [14,21]. Thus we chose to limit the product inhibition by restricting the amount of produced cellobiose to a maximum of 1 mM. The values that we obtained for *p*NP-lac activity are most likely underestimates, since the *K*_i _for cellobiose is 20 μM for the hydrolysis of *p*NP-lac [20], and the cellobiose concentration was higher in most of our experiments.

## Abbreviations

*k*_cat_: catalytic rate constant; *K*_i_: inhibition constant; *K*: binding constant

## Competing interests

MJB and KB are employed by Novozymes A/S (Bagsværd, Denmark), which is a major enzyme producer. PW declares no competing interests.

## Authors' contributions

MJB designed, planned and conducted the study, and drafted the manuscript. KB provided the enzymes and reviewed the manuscript. PW participated in the critical discussion of the results and finalized the manuscript. All authors read and approved the final manuscript.
